# Elevated blood pressure, antihypertensive medications and bone health in the population: revisiting old hypotheses and exploring future research directions

**DOI:** 10.1007/s00198-021-06190-0

**Published:** 2021-10-13

**Authors:** D. Canoy, N. C. Harvey, D. Prieto-Alhambra, C. Cooper, H. E. Meyer, B. O. Åsvold, M. Nazarzadeh, K. Rahimi

**Affiliations:** 1 Deep Medicine, Nuffield Department of Women’s and Reproductive Health, University of Oxford, Hayes House 1F, George St., Oxford, OX1 2BQ UK; 2 NIHR Oxford Biomedical Centre, Oxford University Hospitals NHS Foundation Trust, Oxford, UK; 3 MRC Life Course Epidemiology Unit, University of Southampton, Southampton, UK; 4 NIHR Southampton Biomedical Research Centre, University of Southampton and University Hospital Southampton NHS Foundation Trust, Southampton, UK; 5 Centre for Statistics in Medicine, Nuffield Department of Orthopaedics, Rheumatology and Musculoskeletal Sciences, University of Oxford, Oxford, UK; 6 Department of Medical Informatics, Erasmus University Medical Center, Rotterdam, The Netherlands; 7 Department of Community Medicine and Global Health, Faculty of Medicine, Oslo, Norway; 8 Norwegian Institute of Public Health, Oslo, Norway; 9 Department of Endocrinology, Clinic of Medicine, St. Olav’s Hospital, Trondheim University Hospital, Trondheim, Norway; 10 Department of Public Health and Nursing, K.G. Jebsen Center for Genetic Epidemiology, Norwegian University of Science and Technology, Trondheim, Norway

**Keywords:** Antihypertensive drugs, Blood pressure, Bone fracture, Bone mineral density, Osteoporosis

## Abstract

Blood pressure and bone metabolism appear to share commonalities in their physiologic regulation. Specific antihypertensive drug classes may also influence bone mineral density. However, current evidence from existing observational studies and randomised trials is insufficient to establish causal associations for blood pressure and use of blood pressure–lowering drugs with bone health outcomes, particularly with the risks of osteoporosis and fractures. The availability and access to relevant large-scale biomedical data sources as well as developments in study designs and analytical approaches provide opportunities to examine the nature of the association between blood pressure and bone health more reliably and in greater detail than has ever been possible. It is unlikely that a single source of data or study design can provide a definitive answer. However, with appropriate considerations of the strengths and limitations of the different data sources and analytical techniques, we should be able to advance our understanding of the role of raised blood pressure and its drug treatment on the risks of low bone mineral density and fractures. As elevated blood pressure is highly prevalent and blood pressure–lowering drugs are widely prescribed, even small effects of these exposures on bone health outcomes could be important at a population level.

## Introduction

For some years, a close link between cardiovascular and bone health has been hypothesised [1–5], given that there are similarities in the biological risk factors, lifestyle determinants and demographic profile associated with cardiovascular disease (CVD) and bone health conditions [1, 3, 6–10]. A fall in blood pressure (BP) level and use of antihypertensive drugs have been associated with increased risk of falls particularly in the elderly [11, 12], which may consequently increase the risk of bone fractures. Other pathways that may play a role in the pathophysiology of both atherosclerotic vascular disease and osteoporosis can include alterations in the regulatory mechanisms involved in calcium metabolism and homeostasis, stimulation of inflammatory response, and sympathetic nervous system activation [6–8, 13]. In particular, an association between hypertension and low bone mineral density (BMD) has been suggested [14], possibly as a result of calcium loss observed in people with raised BP [13, 15, 16]. The observation that BP may influence bone health is nothing new as many studies in the past few decades have examined associations between BP and BMD as well as fracture risk [17–21] and between BP-lowering drugs and the risk of osteoporosis and fractures [22–34]. These findings were largely based on observational studies and residual confounding could be an issue, so the likely causal role of BP or use of antihypertensive drugs on bone health outcomes remains to be established. Time trends suggest that age-adjusted mean BP and incidences of osteoporosis and fractures have been improving in some populations [35, 36], but the global burden of these conditions remains high [37–39] perhaps because of a demographic shift towards an ageing population. This ageing demographic trend underscores the importance of understanding the role of raised BP in the aetiology of osteoporosis and its important clinical consequence—bone fractures—as managing elevated BP could play a role in maintaining optimal bone health in the population.

In recent years, large-scale population-based cohorts with detailed clinical assessments, biological measures and genetic data are being used in epidemiologic and clinical investigations. Novel study designs and innovative analytical approaches have been developed, and collaborative studies that involve sharing of biomedical data of study cohorts have become a common practice. These research developments provide an opportunity to revisit hypotheses linking cardiovascular and bone health, and explore ways to answer questions that characterise and establish the causal role of raised BP on bone health outcomes in the population. To share our perspective, we describe some of the evidence linking BP with bone health, identify difficulties in establishing their causal relation from existing evidence and elucidate on research challenges and opportunities to help address these aetiological questions.

## High population burden of osteoporosis and fractures

Osteoporosis is a systemic skeletal disease characterised by low bone mass and deterioration of the microarchitecture of bone tissue, which increases fragility of the bone and susceptibility to fractures [40, 41]. Its prevalence increases with age, and markedly so in women soon after menopause [37, 42, 43]. The bone mineral density (BMD), reported in values relative to the peak bone mass of a standard comparator such as among healthy young women, is used to define osteopenia (BMD T-score of − 2.5 to − 1.0) and osteoporosis (BMD T-score below − 2.5) [44–47]. Worldwide prevalence of low BMD has doubled from 1990 (0.12%) to 2010 (0.21%) and accounted for up to a third of falls-related deaths [38]. Indeed, it has been estimated that 1 in 3 women and 1 in 5 men aged ≥ 50 years will have osteoporosis-related fractures globally [37]. Thus, efforts to reduce the burden of poor bone health are of utmost importance.

## Blood pressure may influence bone health

Elevated BP, or hypertension, is the leading cause of cardiovascular disease morbidity and mortality in many regions worldwide [48]. It is a condition that significantly raises the risks of coronary heart disease, stroke, and renal disease, and also the world’s leading cause of premature death. Over 1.13 billion people globally have elevated BP, with two-thirds of them living in low- and middle-income countries [49]. The prevalence of raised BP increases with age, and in England, it has been estimated that over half of all adults aged ≥ 65 years in 2019 will have had hypertension [50]. There are public health preventive measures to reduce this burden [51]. Moreover, antihypertensive medications are effective, affordable and generally safe, and therefore widely prescribed for managing hypertension in many populations globally. Nevertheless, fewer than 1 in 5 people with hypertension globally have their BP levels under control [48]. In England, nearly a third of all adults have hypertension. More specifically, among all adults in the country, 10% have ‘controlled’ hypertension, 5% have uncontrolled hypertension and 12% have hypertension that remains untreated despite wide access to healthcare provision [50]. However, the continuing efforts for broader use of effective drugs to control and manage raised BP are showing an impact on encouraging trends towards more people with hypertension in England who are receiving treatment and whose raised BP are getting controlled.

### Raised blood pressure, blood pressure reduction and bone health outcomes

While raised BP is an established risk factor of CVD, there are suggestions that it also affects long-term bone health. While the underlying mechanisms are not fully understood, elevated BP has been thought to alter calcium metabolism leading to increased calcium loss [15, 16]. Increased sympathetic nervous system activity, enhanced inflammation response and alteration of parathyroid hormone regulation are pathways that have also been suggested to be involved [1, 6–8, 13]. This relation between raised BP and low bone mineral density could lead to enhanced bone fragility which may increase the risk of fracture, not least in some susceptible individuals such as among the elderly. There is also a suggestion that hypertension affects balance and mobility [52] thereby increasing the likelihood of falls and, consequently, fractures.

Few studies have examined the association between BP and BMD. In one small study, hypertensive women were shown to have lower BMD and higher 24-h urinary calcium excretion than normotensive women [17]. A meta-analysis of observational studies observed a heterogeneity in the association between BP and BMD depending on the anatomical location of the bone and ethnicity, although the study largely included cross-sectional studies [53]. In a prospective investigation based on repeated BMD measurements among 3000 elderly women, elevated BP was associated with increased bone loss in the femoral neck after 3 years of follow-up, independently of hormone replacement therapy and use of BP-lowering drugs [14]. It is uncertain if a similar observation can be seen in elderly men.

Limited data exist for investigating the impact of elevated BP on outcomes involving bone fractures. In a large case–control study, a 27% increased risk of any fracture was associated within 3 years of a diagnosis of hypertension, and 11% increase in risk in the longer term [19]. In a prospective study involving 1032 men and 1701 women aged ≥ 50 years, elevated BP was associated with increased risk of any or hip fracture in women, with similar but less precise estimates in men [21]. In another study, mean arterial pressure or hypertension was not shown to predict incident hip fracture, but the study was based only on 176 events in men and 458 in women, and did not account for the use of antihypertensive treatment [54].

There are also other possible pathways by which elevated BP, or rather, its pharmacologic reduction, can influence bone health. Susceptible individuals may develop syncope or hypotension soon after initiating antihypertensive treatment, leading to injurious falls [12] and, consequently, to fractures [55]. Indeed, a history of a fall is a well-established predictor of future fractures [10]. Three randomised clinical trials (RCTs) with relatively long follow-up have separately reported on the effect of BP reduction on fracture outcomes (Table 1), but these trials only involved less than 500 fracture events collectively [56–58]. One trial [56] reported no difference in the risk of any fracture between comparison groups, while the other two trials [57, 58] showed suggestive reduction in fracture risk in the active BP-lowering treatment arm, but the confidence interval of the risk estimate included the null value. To our knowledge, no randomised trials have examined the effects of pharmacologic lowering of BP on BMD or osteoporosis outcomes.

**Table 1:** Randomised clinical blood pressure–lowering treatment trials with long-term follow-up and have reported on the risk of fracture as an outcome

	**Trial**			
	**ACCORD** [58] (*N* = 3099)	**ALLHAT** [77] (*N* = 22,180)	**HYVET** [57] (*N* = 3845)	**SHEP** [56] (*N* = 4736)
**Study population**	Age ≥ 40 years with diabetes and increased CVD risk	Age ≥ 55 years with hypertension and other CVD risk factors	Age ≥ 80 years with sustained SBP ≥ 160 mmHg	Age ≥ 60 years with hypertension, and no previous treatment
**Comparisons**	More vs less intense treatment	Drug class comparison (thiazide vs ACEI, CCB or ACEI and CCB)	Placebo-controlled	Placebo-controlled
**Drug intervention**	Drug class available in clinical practice	Diuretic (chlorthalidone), CCB (amlodipine), and ACEI (lisinopril)*; additionally with atenolol, clonidine or reserpine if required	Diuretic (indapamide); additionally with ACEI (perindopril) if required	Diuretic (perindopril) and/or diuretic (indapamide)
**Outcome, total *N* (%)**	Non-spine fractures, 270 (8.7)	Hip or pelvic fracture, 338 (1.5)	First fracture, 90 (2.3)	Any fracture, 104 (2.2)
Active group, *N* (%)	116 (7.6)	135 (1.3)	38 (1.5)	57 (2.4)
Control group, *N* (%)	154 (9.8)	203 (1.7)	52 (2.0)	47 (2.0)
**Risk estimate comparing active treatment vs control group**	HR = 0.79 (95% CI 0.62 to 1.01)	HR = 0.78 (95% CI 0.63 to 0.97); HR = 0.75 (95% CI 0.58 to 0.98) if ACEI only as comparator; HR = 0.82 (95% CI 0.63 to 1.08) if CCB only as comparator	HR = 0.69 (95% CI 0.46 to 1.05); HR = 0.58 (95% CI 0.33 to 1.00) if adjusted for baseline predictors of fracture	*z* = 0.8 (*p* = 0.4)
**Comment**	Other site-specific fractures also reported	HR are age- and sex-adjusted; subgroup analysis consistent; post trial to 8 years similar but wide CI	Definite or probable fractures only	

*ACCORD* Action to Control Cardiovascular Risk in Diabetes, *ALLHAT* Antihypertensive and Lipid-Lowering Treatment to Prevent Heart Attacks Trial, *HYVET* Hypertension in the Very Elderly Trial, *SHEP* Systolic Hypertension in the Elderly Program, *ACEI* angiotensin-converting enzyme inhibitor, *CCB* calcium channel blocker, *HR* hazard ratio, *CI* confidence interval

^*^An α-blocker (doxazosin) trial arm was terminated early

### Antihypertensive drug class and bone health outcomes

Other than the unintended effects of antihypertensive medications in lowering BP on falls, others have shown class-specific effects of antihypertensive drugs by affecting different pathways involved in bone remodelling (Table 2). Thiazide diuretics are widely prescribed drugs to manage elevated BP, and this drug can also modulate calcium homeostasis [59]. It has been reported that thiazides can reduce urinary excretion of calcium by 40% [60, 61] as well as stimulate the production of osteoblast differentiation markers and enhance bone calcium uptake by inhibiting thiazide-sensitive sodium chloride cotransporter which are expressed in human osteoblasts [62, 63]. In contrast, loop diuretics are associated with increased urinary calcium excretion and increase parathyroid hormone levels and bone-specific alkaline phosphatase, which could be indicative of accelerated bone remodelling [64, 65]. For both types of diuretics, their use may promote nocturia, which could increase the likelihood of a fall and/or fractures particularly in the elderly. β-Blockers have been suggested to inhibit osteoclastic activity thereby decreasing bone resorption [25, 66]. Selective β-blockers particularly inhibit signalling pathways via β_1_-adrenergic receptors that are expressed in human bones [67]. The renin–angiotensin–aldosterone system not only has systemic effects but also local effects in several tissues including the bone which might explain some of the effects of angiotensin-converting enzyme inhibitors (ACEI) in improving bone mineral density, albeit similar effects are not seen for angiotensin-II receptor blockers (ARB) [59, 68]. There are, therefore, several plausible pathways by which antihypertensive drug classes—collectively or individually—can have an impact on important bone health outcomes.

**Table 2:** Blood pressure–lowering drugs and hypothesised effects and mechanisms on bone health and fracture risk (adapted and modified from Ghosh and Majumdar [59])

**Medications**	**Potential mechanisms affecting bone health**	**Effect on bone mineral density**	**Effect on fracture risk**
**All blood pressure–lowering drugs**	Blood pressure reduction leading to syncope, hypotension and falls	↔	↑
	Reduction in sympathetic nervous system stimulation	↑	↓
**By drug class**			
Thiazide diuretic	Direct stimulation of osteoblasts	↑	↓
	Bone formation	↑	↓
Loop diuretic	Increased urinary calcium loss	↓	↑
	Falls	↔	↑
Spironolactone	Inhibition of aldosterone receptors	↑	↓
β-Blocker	Inhibition of β_2_-adrenergic receptors in osteoblast	↑	↓
ACE-inhibitor	Inhibition of ACE in local RAAS in bone	↑	↓
ARB	Direct blockade for angiotensin-II receptor	↔	↔
CCB	Inhibition of voltage-gated calcium channel	↔	↔
Nitrates	Donates nitric oxide	↑	↓
	Suppression of osteoclast	↑	↓

↑, increase; ↓, decrease; ↔ , probably no discernible impact; *ACE* angiotensin-converting enzyme, *RAAS* renin–angiotensin–aldosterone system, *ARB* angiotensin-II receptor blocker, *CCB* calcium channel blocker

Several observational studies have examined associations of specific BP-lowering drug classes with bone loss or fracture risk [59]. While some findings are inconsistent, many have suggested protective effects on BMD (and reduction in fracture risk) for thiazide diuretic [69–72], β-blocker [26, 73] and ACEI [68, 73, 74]. Loop diuretics have been reported to increase the risk of fracture [27, 70, 75, 76], with no or little evidence of any impact of the use of ARB and calcium channel blocker (CCB) on bone fractures [32, 70, 73, 74, 76]. Evidence from RCTs also remains limited. To date, only one long-term trial has investigated the effects of specific antihypertensive drug class on fracture risk [77]. Involving 22,180 participants and accruing 338 hip or pelvic fractures over 4 years of follow-up, thiazide treatment showed 20% reduction in fracture risk when compared to treatment with ACEI or CCB.

So far, we see that much of the existing findings on BP level or BP-lowering drug classes on bone health outcomes have been based on observational studies, with only a handful using a prospective study design. Since both hypertension and osteoporosis and fractures are influenced by similar factors as age, body size, physical activity level and co-existing chronic conditions, these factors need to be considered and will pose analytical challenges when establishing the causal relation between raised blood pressure and bone health outcomes using data from observational studies. As exposure to these drugs in these studies is mainly based on self-reports, details on timing of prescription as well as the dose and duration of drug treatment are often missing, which limits characterisation of exposure to these drugs. Given how common hypertension is and how widely antihypertensive drugs are being prescribed, even small effects of these exposures could be relevant at the population level. It is therefore crucial to determine the causal relation between BP and BP-lowering drugs with bone health, as it will improve our understanding of the additional implications of BP control in the promotion of optimal bone health. Since the prevalence of raised BP and use of antihypertensive medications increase with age, and those at risk to suffer from metabolic bone disorders and fractures are also more likely to be older, understanding the nature of the associations is important for maximising the benefits and reducing the risks associated with BP control and treatment.

## Advancing research into blood pressure and bone health in the population: current opportunities

Developments in population-based research in recent years have opened up opportunities to address fundamental questions on the role of elevated BP in the aetiology of osteoporosis and fractures. Access to databases providing detailed health data for large numbers of people allows us to investigate associations between BP and bone health outcomes prospectively and with sufficient statistical power. Further, advances in study designs and innovative analytical techniques have drawn us towards making causal inference with more credence. There is also an increasing trend among research communities across disciplines towards working collaboratively and sharing research data and expertise, which have opened up new ways to re-examine old, unanswered questions using novel ideas and perspectives.

### Big data from large-scale cohort studies and healthcare databases

Routinely collected healthcare data have become increasingly an important resource to generate and test hypotheses in clinical research. Anonymised data are extracted from electronic health records (EHRs), such as the United Kingdom (UK) Clinical Practice Research Database [78]. These EHRs provide rich datasets that include time-stamped information on clinical measures, diagnoses and prescriptions and are linked to various national databases that further enrich the datasets to incorporate information on hospitalisations and vital status. These linkages at individual level allow investigations on the prospective associations of hypertension and antihypertensive drug use on bone health outcomes including osteoporosis and fractures. As drug dose and duration can be estimated from these medical records, detailed characterisation of drug exposure is possible to conduct in this context. In the UK, where 97% of the population are registered with the National Health Service, EHRs provide clinical data that are generalisable to the population [79]. Similar possibilities exist in other countries using their national healthcare or prescription databases [80–82]. By linking prescriptions with other administrative health records, it is possible to create anonymised records of prescriptions and relevant health data at individual level.

In addition, several cohorts involving large numbers of participants have collected detailed information on personal characteristics, medical history, lifestyle factors, biological samples, genetic data and clinical measures such as bone densitometry and heel bone ultrasound. For example, in the UK Biobank, these data have been collected for nearly 0.5 million individuals, including calcaneal ultrasound measures for the whole cohort and total dual-energy X-ray absorptiometry in a large subset of the study population [83–85]. Similar detailed phenotypic and genetic data have been collected in other cohorts, such as the population-based Trøndelag Health Study with genetic information combined with phenotypic data that include BMD measurements and prospectively recorded fracture information [86]. Indicators of bone health as well as diagnoses of osteoporosis and fractures are collected in these cohort studies. Data from EHR and well-characterised cohort studies have detailed health information that allows analyses that account for the potential effects of confounding factors. With large numbers of participants and long follow-up, health outcomes will accrue in sufficient numbers and allow conducting stratified analysis to investigate associations in important subgroups, such as by age and sex.

Trials investigating the effects of antihypertensive drugs involving large numbers of participants with relatively long follow-up have been conducted for many years. Findings from these RCTs, particularly by pooling evidence from across these trials, have helped establish the causal role of elevated BP in the aetiology of cardiovascular disease and clearly demonstrated the efficacy of antihypertensive drugs in reducing cardiovascular disease risk [87]. In recent years, collaborative efforts of trialists have allowed pooling of evidence based on individual-level data, which further provided evidence into the efficacy of BP-lowering treatment across important patient subgroups and clinical characteristics [88]. While bone health conditions are not the primary outcomes of these trials, adverse events and other unintended consequences of antihypertensive drug treatments are commonly collected, which may include information on hypotension, falls and fractures. The Blood Pressure Lowering Treatment Trialists’ Collaboration (BPLTTC) (www.bplttc.org) is one such collaboration which recently has been investigating the efficacy and safety of antihypertensive drug treatment [89]. As many of the trials in the collaboration have collected safety data, it could be an important resource to provide randomised evidence for the effects of BP reduction and specific effects of antihypertensive drug classes on fracture risk.

### Methodological and analytical innovations

#### Epidemiological investigations

Designing studies and analysing data to examine associations between an exposure (e.g. raised BP or specific classes of BP-lowering drugs) and an outcome (e.g. low BMD or fracture) in cohort studies require careful consideration as associations based on observational data are prone to biases, confounding and reverse causation. As bone health outcomes are likely to affect the elderly, analysis should account for competing risk such as from other causes of death. While using relevant study designs may help establish temporality of the association of the exposure with the outcome and confounding factors could be adjusted for by employing appropriate statistical methods, residual confounding remains a possibility due to unmeasured or imprecisely measured confounders. When the exposure of interest involves pharmacologic treatments, confounding by indication is an important issue as the clinical indication for the drug treatment in itself may affect the outcome of interest. Some have also suggested that osteoporosis is associated with an increased risk of cardiovascular disease [3, 90], so reverse causation is also a possibility. However, a number of large-scale cohort studies are well-characterised and extensively phenotyped, allowing for potential confounders to be accounted for in the analyses. Data from repeat measurements provide estimates of variability that can be used to correct for imprecision of measurements and adjust for regression dilution [91], an issue particularly relevant for blood pressure [92]. The long follow-up of these cohorts also allows for time-stratified analysis, which permits exploring reverse causality, such as by excluding outcomes occurring during the early years of follow-up. Another important consideration is body weight or body mass index (BMI), which is positively associated with blood pressure and inversely associated with bone density [93, 94] and risk of hip fracture [95–97]. It is therefore important to take into account the impact of BMI when studying the association between BP and bone health.

Analysis using data extracted from EHRs faces similar issues on bias and confounding [98] as analysis using data from observational cohort studies. However, an important issue to consider when using EHR is missing data [99]. Since EHR data are collected primarily for administrative rather than research purpose, information relevant to the study may be missing for many individuals. Thus, to account for these missing data, imputation techniques can be employed to address this problem [99–101]. As EHRs include detailed information on drug prescriptions for a large number in the population, it would be an important resource for addressing questions on the effects of antihypertensive drug classes that are unlikely to be answered reliably by BP-lowering trials, perhaps because some outcomes, such as osteoporosis, take a long time to develop and get diagnosed, and most BP-lowering treatment trials do not actively follow up study participants beyond 5 years [102]. While comparing drug treatment effects without randomisation is prone to bias and confounding, methodological developments in designing studies using EHR could address these potential issues. It has been proposed that a target randomised trial can be emulated using large-scale EHR data, and a framework has been developed to serve as a guide when designing such investigations [103–106]. For example, the effects of statin treatment on cardiovascular disease and cancer tend to show contrasting findings obtained from RCTs and observational studies. However, by designing the study using observational data to ‘mimic’ the target RCT study population and comparison groups, findings from this trial emulation were shown to be consistent with the trial [103]. Using this relatively novel design to conduct studies using EHR data may be useful in this particular context to enable to address questions on BP treatment and bone health that are not ordinarily feasible to be answered using evidence from existing randomised trials.

#### Mendelian randomisation studies

Naturally randomised genetic variants associated with specific phenotypes are increasingly being applied in epidemiologic analysis to investigate unconfounded associations between an exposure and an outcome of interest [107, 108]. This approach is based on Mendel’s second law which follows the principle of the random assortment of alleles during meiosis involving the transfer of deoxyribonucleic acid from parent to offspring during gamete formation. Inheriting a particular genetic variant by an individual is independent of other characteristics. When these individuals are grouped together in the population according to a specific genotype that is associated with a particular phenotype, they should be similar other than for the genetically determined phenotype. If the genetic variant alters or reflects the biological effects of the phenotype, such as BP, the effects of the phenotype can be predicted by the genetic variant for the phenotype. The inheritance of a particular set of alleles could be thought of as a form of naturally occurring randomisation to different levels of exposures. Thus, Mendelian randomisation (MR) is a form of instrumental variable analysis that uses genetic variants as instruments, which could be used in an analysis to diminish issues on confounding and reverse causality in exposure-outcome associations [107, 109]. Multiple single nucleotide polymorphisms (SNPs) associated with a specific phenotype can be combined and used as an instrument in MR analysis [110]. Recent genome-wide association studies (GWAS) have identified over 270 single nucleotide polymorphisms associated with systolic BP in over a million people of European ancestry [111–113]. These variants have been used and validated on coronary heart disease and stroke outcomes and showed findings to be consistent with the randomised evidence from BP-lowering trials [114], suggesting that these SNPs are valid instruments for MR analyses to examine a causal association between BP and bone health outcomes.

In addition, SNPs that encode proteins relating to the functions of specific antihypertensive drug classes have also been identified. For example, selective β-blocker acts by inhibiting the activation of adrenergic receptor β_1_ (ADRβ_1_) which results in reduced myocardial contractility and heart rate leading to a fall in BP [115]. The ADRβ_1_ gene encodes for this receptor which could then be used as a surrogate for exposure to selective β-blockers. Indeed, genetic variants that could be used to evaluate the effects of antihypertensive drug classes, particularly for thiazides, ACEI, ARB, β-blocker and CCB, have been identified [116, 117]. It is, therefore, possible to conduct MR studies to examine associations between specific classes of antihypertensive drugs and bone health outcomes. However, unlike the number of SNPs associated with BP, the number of variants associated with BP-lowering drug classes is limited, and single cohort studies may lack sufficient power to conduct MR analyses in this context. To address this limitation, methods have been developed to allow conducting MR association studies by using summary data from large-scale GWAS without the need for individual-level data at the same time increasing statistical power [118]. In a two-sample MR, the instruments will include all the BP-related variants identified in a separate and independent GWAS, and the outcome will be based on the estimates obtained from the GWAS estimates of bone health outcomes. Given that separate GWAS have been conducted for the exposure (e.g. BP and antihypertensive drug class [111–113]) and for the outcome (e.g. BMD, osteoporosis and fractures) [119–123]), two-sample MR investigations could be conducted. It is worth noting that the UK Biobank contributes genetic data to some of these collaborative studies. Thus, it is important to consider the data being used for exposure (e.g. BP GWAS) are separate from the outcome data [124]. This two-sample MR technique is an approach increasingly being used [114, 125]. Since this analysis does not require individual-level data, it circumvents some of the limitations of genomic data access and sharing.

There are, of course, issues to consider when conducting MR analysis, primarily, the issue of pleiotropy [107, 108, 126]. Although findings remain valid with ‘vertical pleiotropy’, when the association with the phenotype is representing the downstream effects of the genetic variants on the exposure, ‘horizontal pleiotropy’ needs to be considered particularly in settings when multiple variants are being used as instruments. However, there are tools to assess the impact of this bias and still allow calculation of valid causal estimate even when horizontal pleiotropy exists [126, 127]. Additionally, the GWAS on BP phenotype also mainly include participants from Western populations; hence, their generalisability to other populations remains uncertain.

#### Individual participant-level data meta-analysis

As mentioned earlier, there were very few RCTs that reported on the effects of BP-lowering treatment and effects of antihypertensive drug class on risk of fractures. Each individual trial does not have sufficient statistical power to examine the effects of BP-lowering treatment on bone health outcomes. However, several RCTs of BP-lowering treatment have previously collaborated to pool individual participant-level data (IPD) to examine important clinical questions. Since the inception of BPLTTC in 1995, it has provided reliable evidence on the efficacy of BP-lowering drugs in reducing major cardiovascular disease events and mortality [89]. In its current phase, the collaboration includes 52 trials and over 350,000 randomised participants [102]. Many of these trials have routinely collected information on fractures as well as predisposing events, such as syncope, hypotension and falls. As the BPLTTC provides the largest IPD on BP-lowering treatment currently available, this resource offers an opportunity to investigate the effects of pharmacologic effects of BP-lowering on the risk of fractures. There are well-established methods for conducting IPD meta-analysis which could be used to examine the impact of drug treatments to lower BP on bone health outcomes [128, 129]. While it is common to conduct meta-analysis based on published or aggregate data, this method is evidently of limited use because most trials have not reported findings on fracture risk, and, by design, it is not possible to conduct stratified analysis, such as by age and sex.

## Summary

The association between BP and bone health is certainly not a new observation. Yet their plausible biologic link is certainly intriguing and deserves further scrutiny. Given the high prevalence of elevated BP and wide use of BP-lowering drugs, the impact of these exposures on bone health may be important in the population. However, for these associations to have relevance in informing clinical practice and public health policy, the causal nature of the relation between BP and bone health has to be established. It is unlikely that a single source of evidence or study design can provide a definitive answer. For population studies, what is likely needed is to combine epidemiologic, genetic and randomised evidence to provide detailed and nuanced understanding of the importance of controlling BP levels to improve bone health of the population.

## Data Availability

Not applicable.

## References

[1] Doherty TM, Asotra K, Fitzpatrick LA, Qiao JH, Wilkin DJ, Detrano RC, Dunstan CR, Shah PK, Rajavashisth TB (2003). Calcification in atherosclerosis: bone biology and chronic inflammation at the arterial crossroads. Proc Natl Acad Sci U S A.

[2] McFarlane SI, Muniyappa R, Shin JJ, Bahtiyar G, Sowers JR (2004). Osteoporosis and cardiovascular disease: brittle bones and boned arteries, is there a link?. Endocrine.

[3] Farhat GN, Cauley JA (2008). The link between osteoporosis and cardiovascular disease. Clin Cases Miner Bone Metab.

[4] London GM (2011). Soft bone - hard arteries: a link?. Kidney Blood Press Res.

[5] Byon CH, Chen Y (2015). Molecular mechanisms of vascular calcification in chronic kidney disease: the link between bone and the vasculature. Curr Osteoporos Rep.

[6] Singh MV, Chapleau MW, Harwani SC, Abboud FM (2014). The immune system and hypertension. Immunol Res.

[7] Abboud FM, Harwani SC, Chapleau MW (2012). Autonomic neural regulation of the immune system: implications for hypertension and cardiovascular disease. Hypertension.

[8] Elefteriou F (2018). Impact of the autonomic nervous system on the skeleton. Physiol Rev.

[9] Ensrud KE (2013). Epidemiology of fracture risk with advancing age. J Gerontol A Biol Sci Med Sci.

[10] National Institute for Health and Care Excellence (2012) Osteoporosis: assessing the risk of fragility fracture [CG146]. https://www.nice.org.uk/guidance/cg146. Accessed 8 Apr 2021 32186835

[11] Klein D, Nagel G, Kleiner A, Ulmer H, Rehberger B, Concin H, Rapp K (2013). Blood pressure and falls in community-dwelling people aged 60 years and older in the VHM&PP cohort. BMC Geriatr.

[12] Tinetti ME, Han L, Lee DS, McAvay GJ, Peduzzi P, Gross CP, Zhou B, Lin H (2014). Antihypertensive medications and serious fall injuries in a nationally representative sample of older adults. JAMA Intern Med.

[13] Grobbee DE, Hackeng WH, Birkenhager JC, Hofman A (1988). Raised plasma intact parathyroid hormone concentrations in young people with mildly raised blood pressure. Br Med J (Clin Res Ed).

[14] Cappuccio FP, Meilahn E, Zmuda JM, Cauley JA (1999). High blood pressure and bone-mineral loss in elderly white women: a prospective study. Study of Osteoporotic Fractures Research Group. Lancet.

[15] McCarron DA (1982). Low serum concentrations of ionized calcium in patients with hypertension. N Engl J Med.

[16] Cappuccio FP, Kalaitzidis R, Duneclift S, Eastwood JB (2000). Unravelling the links between calcium excretion, salt intake, hypertension, kidney stones and bone metabolism. J Nephrol.

[17] Tsuda K, Nishio I, Masuyama Y (2001). Bone mineral density in women with essential hypertension. Am J Hypertens.

[18] Perez-Castrillon JL, Martin-Escudero JC, Alvarez Manzanares P, Cortes Sancho R, Iglesias Zamora S, Garcia Alonso M (2005). Hypertension as a risk factor for hip fracture. Am J Hypertens.

[19] Vestergaard P, Rejnmark L, Mosekilde L (2009). Hypertension is a risk factor for fractures. Calcif Tissue Int.

[20] El-Bikai R, Tahir MR, Tremblay J (2015). Association of age-dependent height and bone mineral density decline with increased arterial stiffness and rate of fractures in hypertensive individuals. J Hypertens.

[21] Yang S, Nguyen ND, Center JR, Eisman JA, Nguyen TV (2014). Association between hypertension and fragility fracture: a longitudinal study. Osteoporos Int.

[22] Jones G, Nguyen T, Sambrook PN, Eisman JA (1995). Thiazide diuretics and fractures: can meta-analysis help?. J Bone Miner Res.

[23] Nguyen TV, Eisman JA, Kelly PJ, Sambrook PN (1996). Risk factors for osteoporotic fractures in elderly men. Am J Epidemiol.

[24] Herings RM, Stricker BH, de Boer A, Bakker A, Sturmans F, Stergachis A (1996). Current use of thiazide diuretics and prevention of femur fractures. J Clin Epidemiol.

[25] Reid IR (2008). Effects of beta-blockers on fracture risk. J Musculoskelet Neuronal Interact.

[26] Toulis KA, Hemming K, Stergianos S, Nirantharakumar K, Bilezikian JP (2014). Beta-adrenergic receptor antagonists and fracture risk: a meta-analysis of selectivity, gender, and site-specific effects. Osteoporos Int.

[27] Lim LS, Fink HA, Kuskowski MA, Taylor BC, Schousboe JT, Ensrud KE, Osteoporotic Fractures in Men Study G (2008). Loop diuretic use and increased rates of hip bone loss in older men: the Osteoporotic Fractures in Men Study. Arch Intern Med.

[28] Buford TW (2016). Hypertension and aging. Ageing Res Rev.

[29] Kruse C, Eiken P, Vestergaard P (2016). Optimal age of commencing and discontinuing thiazide therapy to protect against fractures. Osteoporos Int.

[30] Solomon DH, Ruppert K, Zhao Z, Lian YJ, Kuo IH, Greendale GA, Finkelstein JS (2016). Bone mineral density changes among women initiating blood pressure lowering drugs: a SWAN cohort study. Osteoporos Int.

[31] Weiss J, Freeman M, Low A, Fu R, Kerfoot A, Paynter R, Motu’apuaka M, Kondo K, Kansagara D,  (2017). Benefits and harms of intensive blood pressure treatment in adults aged 60 years or older: a systematic review and meta-analysis. Ann Intern Med.

[32] Kunutsor SK, Blom AW, Whitehouse MR, Kehoe PG, Laukkanen JA (2017). Renin-angiotensin system inhibitors and risk of fractures: a prospective cohort study and meta-analysis of published observational cohort studies. Eur J Epidemiol.

[33] Barzilay JI, Davis BR, Pressel SL, Ghosh A, Puttnam R, Margolis KL, Whelton PK (2017). The impact of antihypertensive medications on bone mineral density and fracture risk. Curr Cardiol Rep.

[34] Sommerauer C, Kaushik N, Woodham A, Renom-Guiteras A, Martinez YV, Reeves D, Kunnamo I, Al Qur An T, Hubner S, Sonnichsen A (2017). Thiazides in the management of hypertension in older adults - a systematic review. BMC Geriatr.

[35] N. C. D. Risk Factor Collaboration (2017). Worldwide trends in blood pressure from 1975 to 2015: a pooled analysis of 1479 population-based measurement studies with 19.1 million participants. Lancet.

[36] Concin H, Brozek W, Benedetto KP (2016). Hip fracture incidence 2003–2013 and projected cases until 2050 in Austria: a population-based study. Int J Public Health.

[37] Cooper C, Ferrari S, on behalf of the IOF Board and Executive Committee (2019) IOF Compendium of osteoporosis. International Osteoporosis Foundation. https://www.osteoporosis.foundation/sites/iofbonehealth/files/2020-01/IOF-Compendium-of-Osteoporosis-web-V02.pdf: Accessed 12 April 2021

[38] Sanchez-Riera L, Carnahan E, Vos T (2014). The global burden attributable to low bone mineral density. Ann Rheum Dis.

[39] Borgstrom F, Karlsson L, Ortsater G (2020). Fragility fractures in Europe: burden, management and opportunities. Arch Osteoporos.

[40] (1991) Consensus development conference: prophylaxis and treatment of osteoporosis. Am J Med 90:107–110 10.1016/0002-9343(91)90512-v 1986575

[41] (1993) Consensus development conference: diagnosis, prophylaxis, and treatment of osteoporosis. Am J Med 94:646–650 10.1016/0002-9343(93)90218-e 8506892

[42] Kanis JA, Oden A, McCloskey EV, Johansson H, Wahl DA, Cooper C, Epidemiology IOFWGo, Quality of L (2012). A systematic review of hip fracture incidence and probability of fracture worldwide. Osteoporos Int.

[43] Gourlay ML, Overman RA, Ensrud KE (2015). Bone density screening and re-screening in postmenopausal women and older men. Curr Osteoporos Rep.

[44] Kanis JA, Melton LJ, Christiansen C, Johnston CC, Khaltaev N (1994). The diagnosis of osteoporosis. J Bone Miner Res.

[45] (1994) Assessment of fracture risk and its application to screening for postmenopausal osteoporosis. World Health Organization, Geneva 7941614

[46] World Health Organization (2003) Prevention and management of osteoporosis. World Health Organization. https://apps.who.int/iris/bitstream/handle/10665/42841/WHO_TRS_921.pdf;jsessionid=D9C403A50A7262764ACA8573D859A58C?sequence=1. Accessed 4 March 2021

[47] Kanis JA, Adachi JD, Cooper C (2013). Standardising the descriptive epidemiology of osteoporosis: recommendations from the Epidemiology and Quality of Life Working Group of IOF. Osteoporos Int.

[48] GBD 2019 Risk Factors Collaborators (2020). Global burden of 87 risk factors in 204 countries and territories, 1990–2019: a systematic analysis for the Global Burden of Disease Study 2019. Lancet.

[49] World Health Organization (2019) Hypertension. https://www.who.int/news-room/fact-sheets/detail/hypertension. Accessed 20 Jun 2021

[50] Fat LN (2020) Health Survey for England 2019 Adults’ health. Centre HaSCI. https://digital.nhs.uk/data-and-information/publications/statistical/health-survey-for-england/2019. Accessed 12 April 2021

[51] National Health Service Prevention. High blood pressure (hypertension). https://www.nhs.uk/conditions/high-blood-pressure-hypertension/prevention/. Accessed 12 April 2021

[52] Rosano C, Longstreth WT, Boudreau R, Taylor CA, Du Y, Kuller LH, Newman AB (2011). High blood pressure accelerates gait slowing in well-functioning older adults over 18-years of follow-up. J Am Geriatr Soc.

[53] Ye C, Xu M, Wang S, Jiang S, Chen X, Zhou X, He R (2016). Decreased bone mineral density is an independent predictor for the development of atherosclerosis: a systematic review and meta-analysis. PLoS One.

[54] Dominic E, Brozek W, Peter RS, Fromm E, Ulmer H, Rapp K, Concin H, Nagel G (2020). Metabolic factors and hip fracture risk in a large Austrian cohort study. Bone Rep.

[55] Butt DA, Mamdani M, Austin PC, Tu K, Gomes T, Glazier RH (2012). The risk of hip fracture after initiating antihypertensive drugs in the elderly. Arch Intern Med.

[56] SHEP Cooperative Research Group (1991). Prevention of stroke by antihypertensive drug treatment in older persons with isolated systolic hypertension. Final results of the Systolic Hypertension in the Elderly Program (SHEP). JAMA.

[57] Peters R, Beckett N, Burch L, de Vernejoul MC, Liu L, Duggan J, Swift C, Gil-Extremera B, Fletcher A, Bulpitt C (2010). The effect of treatment based on a diuretic (indapamide) +/- ACE inhibitor (perindopril) on fractures in the Hypertension in the Very Elderly Trial (HYVET). Age Ageing.

[58] Margolis KL, Palermo L, Vittinghoff E (2014). Intensive blood pressure control, falls, and fractures in patients with type 2 diabetes: the ACCORD trial. J Gen Intern Med.

[59] Ghosh M, Majumdar SR (2014). Antihypertensive medications, bone mineral density, and fractures: a review of old cardiac drugs that provides new insights into osteoporosis. Endocrine.

[60] Wang XY, Masilamani S, Nielsen J, Kwon TH, Brooks HL, Nielsen S, Knepper MA (2001). The renal thiazide-sensitive Na-Cl cotransporter as mediator of the aldosterone-escape phenomenon. J Clin Invest.

[61] Obermuller N, Bernstein P, Velazquez H, Reilly R, Moser D, Ellison DH, Bachmann S (1995). Expression of the thiazide-sensitive Na-Cl cotransporter in rat and human kidney. Am J Physiol.

[62] Barry EL, Gesek FA, Kaplan MR, Hebert SC, Friedman PA (1997). Expression of the sodium-chloride cotransporter in osteoblast-like cells: effect of thiazide diuretics. Am J Physiol.

[63] Dvorak MM, De Joussineau C, Carter DH, Pisitkun T, Knepper MA, Gamba G, Kemp PJ, Riccardi D (2007). Thiazide diuretics directly induce osteoblast differentiation and mineralized nodule formation by interacting with a sodium chloride co-transporter in bone. J Am Soc Nephrol.

[64] Elmgreen J, Tougaard L, Leth A, Christensen MS (1980). Elevated serum parathyroid hormone concentration during treatment with high ceiling diuretics. Eur J Clin Pharmacol.

[65] Rejnmark L, Vestergaard P, Heickendorff L, Andreasen F, Mosekilde L (2005). Effects of long-term treatment with loop diuretics on bone mineral density, calcitropic hormones and bone turnover. J Intern Med.

[66] Togari A, Arai M, Kondo A (2005). The role of the sympathetic nervous system in controlling bone metabolism. Expert Opin Ther Targets.

[67] Khosla S, Drake MT, Volkman TL, Thicke BS, Achenbach SJ, Atkinson EJ, Joyner MJ, Rosen CJ, Monroe DG, Farr JN (2018). Sympathetic beta1-adrenergic signaling contributes to regulation of human bone metabolism. J Clin Invest.

[68] Shimizu H, Nakagami H, Osako MK, Hanayama R, Kunugiza Y, Kizawa T, Tomita T, Yoshikawa H, Ogihara T, Morishita R (2008). Angiotensin II accelerates osteoporosis by activating osteoclasts. FASEB J.

[69] Reid IR, Ames RW, Orr-Walker BJ, Clearwater JM, Horne AM, Evans MC, Murray MA, McNeil AR, Gamble GD (2000). Hydrochlorothiazide reduces loss of cortical bone in normal postmenopausal women: a randomized controlled trial. Am J Med.

[70] Bokrantz T, Schioler L, Bostrom KB, Kahan T, Mellstrom D, Ljungman C, Hjerpe P, Hasselstrom J, Manhem K (2020). Antihypertensive drug classes and the risk of hip fracture: results from the Swedish primary care cardiovascular database. J Hypertens.

[71] Wasnich RD, Benfante RJ, Yano K, Heilbrun L, Vogel JM (1983). Thiazide effect on the mineral content of bone. N Engl J Med.

[72] Wasnich R, Davis J, Ross P, Vogel J (1990). Effect of thiazide on rates of bone mineral loss: a longitudinal study. BMJ.

[73] Rejnmark L, Vestergaard P, Mosekilde L (2006). Treatment with beta-blockers, ACE inhibitors, and calcium-channel blockers is associated with a reduced fracture risk: a nationwide case-control study. J Hypertens.

[74] Kwok T, Leung J, Barrett-Connor E, Osteoporotic Fractures in Men Research G (2017). ARB users exhibit a lower fracture incidence than ACE inhibitor users among older hypertensive men. Age Ageing.

[75] Rejnmark L, Vestergaard P, Mosekilde L (2006). Fracture risk in patients treated with loop diuretics. J Intern Med.

[76] Corrao G, Mazzola P, Monzio Compagnoni M, Rea F, Merlino L, Annoni G, Mancia G (2015). Antihypertensive medications, loop diuretics, and risk of hip fracture in the elderly: a population-based cohort study of 81,617 Italian patients newly treated between 2005 and 2009. Drugs Aging.

[77] Puttnam R, Davis BR, Pressel SL (2017). Association of 3 different antihypertensive medications with hip and pelvic fracture risk in older adults: secondary analysis of a randomized clinical trial. JAMA Intern Med.

[78] (2020) Clinical Practice Research Datalink. Crown Copyright. https://www.cprd.com/. Accessed 21 Jun 2021

[79] Herrett E, Gallagher AM, Bhaskaran K, Forbes H, Mathur R, van Staa T, Smeeth L (2015). Data resource profile: Clinical Practice Research Datalink (CPRD). Int J Epidemiol.

[80] Bakken IJ, Ariansen AMS, Knudsen GP, Johansen KI, Vollset SE (2020). The Norwegian Patient Registry and the Norwegian Registry for Primary Health Care: research potential of two nationwide health-care registries. Scand J Public Health.

[81] Norwegian Prescription Database (NorPD). Norwegian Institute of Public Health. https://www.fhi.no/en/hn/health-registries/norpd/. Accessed 21 Jun 2021

[82] Pottegard A, Schmidt SAJ, Wallach-Kildemoes H, Sorensen HT, Hallas J, Schmidt M (2017). Data resource profile: the Danish National Prescription Registry. Int J Epidemiol.

[83] Harvey NC, Matthews P, Collins R, Cooper C, Group UKBMA (2013). Osteoporosis epidemiology in UK Biobank: a unique opportunity for international researchers. Osteoporos Int.

[84] Littlejohns TJ, Holliday J, Gibson LM (2020). The UK Biobank imaging enhancement of 100,000 participants: rationale, data collection, management and future directions. Nat Commun.

[85] Sudlow C, Gallacher J, Allen N (2015). UK Biobank: an open access resource for identifying the causes of a wide range of complex diseases of middle and old age. PLoS Med.

[86] Krokstad S, Langhammer A, Hveem K, Holmen TL, Midthjell K, Stene TR, Bratberg G, Heggland J, Holmen J (2013). Cohort Profile: the HUNT Study, Norway. Int J Epidemiol.

[87] Ettehad D, Emdin CA, Kiran A, Anderson SG, Callender T, Emberson J, Chalmers J, Rodgers A, Rahimi K (2016). Blood pressure lowering for prevention of cardiovascular disease and death: a systematic review and meta-analysis. Lancet.

[88] Blood Pressure Lowering Treatment Trialists’ Collaboration (2021). Pharmacological blood pressure lowering for primary and secondary prevention of cardiovascular disease across different levels of blood pressure: an individual participant-level data meta-analysis. Lancet.

[89] Rahimi K, Canoy D, Nazarzadeh M, Salimi-Khorshidi G, Woodward M, Teo K, Davis BR, Chalmers J, Pepine CJ, Blood Pressure Lowering Treatment Trialists Collaboration (2019). Investigating the stratified efficacy and safety of pharmacological blood pressure-lowering: an overall protocol for individual patient-level data meta-analyses of over 300 000 randomised participants in the new phase of the Blood Pressure Lowering Treatment Trialists’ Collaboration (BPLTTC). BMJ Open.

[90] Park J, Yoon YE, Kim KM, Hwang IC, Lee W, Cho GY (2021) Prognostic value of lower bone mineral density in predicting adverse cardiovascular disease in Asian women. Heart 107:1040–1046 10.1136/heartjnl-2020-318764 33963047

[91] Hutcheon JA, Chiolero A, Hanley JA (2010). Random measurement error and regression dilution bias. BMJ.

[92] Ayala Solares JR, Canoy D, Raimondi FED (2019). Long-term exposure to elevated systolic blood pressure in predicting incident cardiovascular disease: evidence from large-scale routine electronic health records. J Am Heart Assoc.

[93] Felson DT, Zhang Y, Hannan MT, Anderson JJ (1993). Effects of weight and body mass index on bone mineral density in men and women: the Framingham study. J Bone Miner Res.

[94] Reid IR, Ames R, Evans MC, Sharpe S, Gamble G, France JT, Lim TM, Cundy TF (1992). Determinants of total body and regional bone mineral density in normal postmenopausal women–a key role for fat mass. J Clin Endocrinol Metab.

[95] De Laet C, Kanis JA, Oden A (2005). Body mass index as a predictor of fracture risk: a meta-analysis. Osteoporos Int.

[96] Armstrong ME, Spencer EA, Cairns BJ, Banks E, Pirie K, Green J, Wright FL, Reeves GK, Beral V, Million Women Study C (2011). Body mass index and physical activity in relation to the incidence of hip fracture in postmenopausal women. J Bone Miner Res.

[97] Johansson H, Kanis JA, Oden A (2014). A meta-analysis of the association of fracture risk and body mass index in women. J Bone Miner Res.

[98] Goldstein BA, Bhavsar NA, Phelan M, Pencina MJ (2016). Controlling for informed presence bias due to the number of health encounters in an electronic health record. Am J Epidemiol.

[99] Haneuse S, Arterburn D, Daniels MJ (2021). Assessing missing data assumptions in EHR-based studies: a complex and underappreciated task. JAMA Netw Open.

[100] Wells BJ, Chagin KM, Nowacki AS, Kattan MW (2013). Strategies for handling missing data in electronic health record derived data. EGEMS (Wash DC).

[101] Beaulieu-Jones BK, Lavage DR, Snyder JW, Moore JH, Pendergrass SA, Bauer CR (2018). Characterizing and managing missing structured data in electronic health records: data analysis. JMIR Med Inform.

[102] Canoy D, Copland E, Nazarzadeh M et al (2021) Effect of antihypertensive drug treatment on long-term blood pressure reduction: an individual patient-level data meta-analysis of 352,744 participants from 51 large-scale randomised clinical trials. medRxiv. 10.1101/2021.02.19.21252066

[103] Danaei G, Rodriguez LA, Cantero OF, Logan R, Hernan MA (2013). Observational data for comparative effectiveness research: an emulation of randomised trials of statins and primary prevention of coronary heart disease. Stat Methods Med Res.

[104] Hernan MA, Robins JM (2016). Using big data to emulate a target trial when a randomized trial is not available. Am J Epidemiol.

[105] Hernan MA, Sauer BC, Hernandez-Diaz S, Platt R, Shrier I (2016). Specifying a target trial prevents immortal time bias and other self-inflicted injuries in observational analyses. J Clin Epidemiol.

[106] Dickerman BA, Garcia-Albeniz X, Logan RW, Denaxas S, Hernan MA (2019). Avoidable flaws in observational analyses: an application to statins and cancer. Nat Med.

[107] Davey Smith G, Hemani G (2014). Mendelian randomization: genetic anchors for causal inference in epidemiological studies. Hum Mol Genet.

[108] Bennett DA, Holmes MV (2017). Mendelian randomisation in cardiovascular research: an introduction for clinicians. Heart.

[109] Burgess S, Small DS, Thompson SG (2017). A review of instrumental variable estimators for Mendelian randomization. Stat Methods Med Res.

[110] Burgess S, Foley CN, Allara E, Staley JR, Howson JMM (2020). A robust and efficient method for Mendelian randomization with hundreds of genetic variants. Nat Commun.

[111] Ehret GB, Munroe PB, International Consortium for Blood Pressure Genome-Wide Association Studies (2011). Genetic variants in novel pathways influence blood pressure and cardiovascular disease risk. Nature.

[112] Ehret GB, Ferreira T, Chasman DI (2016). The genetics of blood pressure regulation and its target organs from association studies in 342,415 individuals. Nat Genet.

[113] Evangelou E, Warren HR, Mosen-Ansorena D (2018). Genetic analysis of over 1 million people identifies 535 new loci associated with blood pressure traits. Nat Genet.

[114] Nazarzadeh M, Pinho-Gomes AC, Bidel Z, Canoy D, Dehghan A, Smith Byrne K, Bennett DA, Smith GD, Rahimi K (2021). Genetic susceptibility, elevated blood pressure, and risk of atrial fibrillation: a Mendelian randomization study. Genome Med.

[115] Lewis CJ, Gong H, Brown MJ, Harding SE (2004). Overexpression of beta 1-adrenoceptors in adult rat ventricular myocytes enhances CGP 12177A cardiostimulation: implications for ‘putative’ beta 4-adrenoceptor pharmacology. Br J Pharmacol.

[116] Gill D, Georgakis MK, Koskeridis F (2019). Use of genetic variants related to antihypertensive drugs to inform on efficacy and side effects. Circulation.

[117] Walker VM, Kehoe PG, Martin RM, Davies NM (2020). Repurposing antihypertensive drugs for the prevention of Alzheimer’s disease: a Mendelian randomization study. Int J Epidemiol.

[118] Pierce BL, Burgess S (2013). Efficient design for Mendelian randomization studies: subsample and 2-sample instrumental variable estimators. Am J Epidemiol.

[119] Estrada K, Styrkarsdottir U, Evangelou E (2012). Genome-wide meta-analysis identifies 56 bone mineral density loci and reveals 14 loci associated with risk of fracture. Nat Genet.

[120] Zheng HF, Forgetta V, Hsu YH (2015). Whole-genome sequencing identifies EN1 as a determinant of bone density and fracture. Nature.

[121] Kemp JP, Morris JA, Medina-Gomez C (2017). Identification of 153 new loci associated with heel bone mineral density and functional involvement of GPC6 in osteoporosis. Nat Genet.

[122] Medina-Gomez C, Kemp JP, Trajanoska K (2018). Life-course genome-wide association study meta-analysis of total body BMD and assessment of age-specific effects. Am J Hum Genet.

[123] Morris JA, Kemp JP, Youlten SE (2019). An atlas of genetic influences on osteoporosis in humans and mice. Nat Genet.

[124] Burgess S, Davies NM, Thompson SG (2016). Bias due to participant overlap in two-sample Mendelian randomization. Genet Epidemiol.

[125] Nazarzadeh M, Pinho-Gomes AC, Bidel Z (2020). Plasma lipids and risk of aortic valve stenosis: a Mendelian randomization study. Eur Heart J.

[126] Zheng J, Frysz M, Kemp JP, Evans DM, Davey Smith G, Tobias JH (2019). Use of Mendelian randomization to examine causal inference in osteoporosis. Front Endocrinol (Lausanne).

[127] Burgess S, Thompson SG (2017). Interpreting findings from Mendelian randomization using the MR-Egger method. Eur J Epidemiol.

[128] Debray TP, Moons KG, van Valkenhoef G, Efthimiou O, Hummel N, Groenwold RH, Reitsma JB, GetReal Methods Review G (2015). Get real in individual participant data (IPD) meta-analysis: a review of the methodology. Res Synth Methods.

[129] Higgins JP, Whitehead A, Turner RM, Omar RZ, Thompson SG (2001). Meta-analysis of continuous outcome data from individual patients. Stat Med.

